# Increased interest with the introduction of fast-track diagnostic pathway is associated with the regionally increased frequency of giant cell arteritis in Poland: a study based on POLVAS registry data

**DOI:** 10.3389/fmed.2024.1440725

**Published:** 2024-08-02

**Authors:** Marcin Milchert, Krzysztof Wójcik, Jacek Musiał, Anna Masiak, Maria Majdan, Radoslaw Jeleniewicz, Witold Tłustochowicz, Joanna Kur-Zalewska, Małgorzata Wisłowska, Anna Lewandowska-Polak, Joanna Makowska, Marek Brzosko

**Affiliations:** ^1^Department of Rheumatology, Internal Diseases, Diabetology, Geriatrics and Clinical Immunology with Gastroenterology Department, Pomeranian Medical University, Szczecin, Poland; ^2^Department of Gastroenterology, Pomeranian Medical University, Szczecin, Poland; ^3^2nd Department of Internal Medicine, Faculty of Medicine, Jagiellonian University Medical College, Kraków, Poland; ^4^Department of Internal Medicine, Connective Tissue Diseases and Geriatrics, Medical University of Gdańsk, Gdańsk, Poland; ^5^Department of Rheumatology and Connective Tissue Diseases, Medical University of Lublin, Lublin, Poland; ^6^Military Medicine Institute, Warsaw, Poland; ^7^Clinical Trials Support Center, Military Institute of Medicine - National Research Institute, Warsaw, Poland; ^8^National Institute of Geriatrics, Rheumatology and Rehabilitation, Warsaw, Poland; ^9^Department of Rheumatology, Medical University of Lodz, Lodz, Poland

**Keywords:** giant cell arteritis, fast-track clinic, ultrasound, prevalence, registry

## Abstract

Slavic populations, such as those in Poland, are considered to have a low prevalence of giant cell arteritis (GCA), although epidemiological data are sparse. The study aimed to compare the reported frequency of GCA in various regions of Poland and analyze the differences between them. We conducted a multicenter, retrospective study of all GCA patients included in the POLVAS registry—the first large multicenter database of patients with vasculitis in Poland. The data from the POLVAS registry were compared with the reported prevalence provided by national insurers from the corresponding regions. A 10-fold increase in the diagnostic rates of GCA was observed in Poland between 2008 and 2019, reaching 8.38 per 100,000 population > 50 years old. It may be attributed to increased interest accompanied by improved diagnostic modalities with the introduction of ultrasound-based, fast-track diagnostic pathways in some centers. However, regional inequities are present, resulting in 10-fold differences (from 2.57 to 24.92) in reported prevalence between different regions. Corticosteroid (CS) monotherapy was the main stem of treatment. Further cooperation and education are needed to minimize regional inequities. This observational study suggests some potential for further increase of the recognizability of GCA and wider use of other than CS monotherapy treatment regimens. We hope that the Polish experience might be interesting and serve as some guidance for the populations where GCA is underdiagnosed.

## Introduction

GCA is the most common vasculitis affecting large- and medium-sized arteries (1). Overall GCA incidence seems not to be increasing, as demonstrated in the homogeneous population of Norway (2). However, some studies demonstrated an increase in the prevalence of GCA that their authors attribute to increased interest and raising awareness of this disease, as demonstrated by the study from northern Germany performed between 1994 and 2006 (3). In this regard, in the Lugo region of Norwest Spain, a progressive increase in the incidence was observed. With respect to this, while the incidence of biopsy-proven GCA was 6.0 per 100,000 people aged 50 years and older from 1981 to 1990, the annual incidence rate in the same region increased up to 15.90 between 1996 and 2000 (4). Therefore, there may still be potential for diagnostic improvement. Clinical phenotypes and outcomes of GCA patients may differ depending on geographic area and ethnicity. In contrast with the above-mentioned Scandinavian and German cohorts, a Slavic population such as this in Poland is not considered to have a high frequency of GCA, although epidemiological data are sparse. A low incidence of GCA may further limit its recognizability. Modern diagnostic techniques such as arterial ultrasound for large vessel vasculitis are mandatory for efficient fast-track clinics to improve the diagnosis of GCA (5). However, the introduction of arterial ultrasound into rheumatology’s daily practice may be troublesome, and time and education are still needed for it to be widely used. An increase in GCA recognizability requires not only the availability of sensitive diagnostic methods (optimally reaching out to reference centers) but also diagnostic awareness, defined as an efficient system for selecting patients suspected of GCA and referring them to undergo specific examinations. Despite advances in the development of new possibilities for treatment, underdiagnosis of GCA can result in serious complications for the patient, with mostly feared but still observed irreversible vision loss (6). Therefore, prompt diagnosis of this disease and timely treatment initiation remain crucial.

This study aimed to compare the reported frequency of GCA in various regions of Poland and to analyze factors potentially influencing observed differences based on the data from the POLVAS registry as interpreted by experienced researchers and practitioners. Such an analysis may uncover potential care problems to improve them.

## Methods

We conducted a multicenter, retrospective study of all GCA patients included in the POLVAS registry (7) before the COVID-19 pandemic, between 2008 and 2019. Patients’ data were supplied by 11 referral centers participating in the POLVAS project from nine administrative regions (Voivodeships), encompassing 70% of the Polish population (27×10^6^ inhabitants). Data were obtained cumulatively, including information on demographics, clinical, laboratory, imaging, pathology, and treatment details, that were collected according to the common protocol. Data from two centers reporting <4 cases were excluded from the analysis, but the reported prevalence of GCA in these regions was calculated. Only patients who met the American College of Rheumatology (ACR) classification criteria for GCA or fulfilled requirements for the nomenclature of GCA according to CHCC 2012 (8) were included in the study. In case of any diagnostic uncertainty as judged by the treating physician, the center was left free to note if the diagnosis was either certain or probable.

We analyzed data on GCA cases reported to the Polish National Insurance Fund (NFZ) from 2008 to 2019 based on ICD10 codes (M31.5 and M31.6). The national insurer is responsible for all of the health care participants in Poland, with the exception of the small number of non-insurance subjects. The data on reported prevalence in individual regions according to national insurer data were compared to the POLVAS registry data (if they were available) from the same regions. By exchanging information about local diagnostic capabilities and management strategies among POLVAS members, we analyzed factors potentially influencing the increase in GCA recognizability in individual regions. The pre-COVID-19 pandemic period was analyzed to avoid difficulties in interpreting data during a potential deepening of healthcare inequities.

The study described in this article has been carried out in accordance with the Code of Ethics of the World Medical Association (Declaration of Helsinki) for experiments involving humans. The study protocol was approved by the Bioethical Commission of Jagiellonian University, decision No 122.6120.25.2016. The ethics committee of each partner has approved the research protocol. Informed consent has been obtained from each subject (or their legally authorized representative).

### Statistical analysis

Descriptive statistics were utilized to analyze trends in GCA diagnosis and management in Poland from the practitioners’ perspective. Categorical data were summarized as percentages. Continuous variables were presented as mean. Calculations and principal component analysis (PCA) were performed using RStudio (version 3.6.0).

## Results

### Demographic data analysis of GCA patients

In 2008, there were 109 GCA cases among Polish patients reported to national insurance institutions, compared to 1,005 cases in 2019 (including both inpatient and outpatient care). The average reported prevalence in 2019 was 8.38 per 100,000 population > 50 years old, but it ranged from 24.92 to 2.57 in different Voivodships (Table 1). A total of 219 patients were included in the POLVAS registry until 2019. All patients were Caucasian. The male-to-female ratio was 1:2. Demographic data of patients are given in Table 2.

**Table 1:** Giant cell arteritis cases reported yearly to national insurance by region and factors demonstrating local interest in the disease.

	Voivodship name (name in polish)*	Population in mln (in 2015)	GCA cases reported to insurance by year												Number of cases reported per region’s population in 2019**	GCA cases reported to the POLVAS registry until 2019	Active fast-track clinics (year of introduction)	Founding sites of POLVAS registry	Attendance at GCA ultrasound courses***
			2008	2009	2010	2011	2012	2013	2014	2015	2016	2017	2018	2019					
1.	West Pomeranian (zachodniopomorskie)	1,71	10	19	12	15	25	32	46	51	72	98	115	133	24,92	48	Yes (2008)	Yes	Yes
2.	Pomeranian (pomorskie)	2,31	5	7	6	31	40	45	43	51	58	56	88	110	15,28	67	Yes (2015)	Yes	Yes
3.	Masovian (mazowieckie) capital city region	5,35	44	50	55	74	87	89	106	116	124	123	153	193	11,56	43	–	Yes	–
4.	Lower Silesian (dolnośląskie)	2,90	9	15	20	18	20	24	34	44	39	45	38	94	10,37	1	–	Yes	Yes
5.	Kuyavian-Pomeranian (kujawsko-pomorskie)	2,09	6	6	3	9	14	17	21	24	29	39	53	60	9,22	4	–	–	–
6.	Lesser Poland (małopolskie)	3,37	2	2	6	16	19	22	25	25	22	24	71	94	8,93	6	Yes (2018)	Yes	–
7.	Świętokrzyskie	1,26	0	2	0	2	6	10	15	11	22	21	19	32	8,16	0	–	–	–
8.	Łódź (łódzkie)	2,49	3	3	1	5	5	6	6	3	8	21	32	51	6,56	14	–	–	–
9.	Subcarpathian (podkarpackie)	2,13	5	3	5	4	10	15	14	14	16	16	29	43	6,48	0	–	–	–
10.	Lublin (lubelskie)	2,14	4	4	2	2	9	9	16	17	19	24	30	40	5,99	35	–	Yes	Yes
11.	Podlaskie (podlaskie)	1,19	3	5	10	7	37	32	20	20	22	28	19	21	5,66	0	–	–	–
12.	Lubusz (lubuskie)	1,02	7	0	1	0	0	1	3	9	6	6	12	14	4,41	0	–	–	–
13.	Greater Poland (wielkopolskie)	3,48	2	9	7	6	12	11	11	15	24	28	33	45	4,15	0	–	–	–
14.	Warmian-Masurian (warmińsko-mazurskie)	1,44	1	1	1	2	3	5	6	5	10	9	15	18	4,01	0	–	–	–
15.	Silesian (śląskie)	4,57	7	10	5	10	14	24	16	23	29	41	44	53	3,72	1	–	Yes	–
16.	Opole (opolskie)	1,00	1	0	4	5	3	5	2	5	2	9	7	8	2,57	0	–	–	–
	All	38,44	109	136	138	206	304	347	384	433	502	469	758	1,005	8,38	219			

GCA – giant cell arteritis. *Order by number of cases reported per region’s population in 2019, **per 100,000 region’s population > 50 years old, ***At least 2 ×10^6^ of population attendees of GCA ultrasound courses organized annually in West Pomeranian from 2013 to 2017.

**Table 2:** Demographic and clinical characteristics of giant cell arteritis patients in the POLVAS registry by different centers.

	Voivodship name (location of its reference center)	*N*	Female *N* (%)	Age at diagnosis (mean ± SD)	Ophthalmological manifestations N (%)	PMR manifestations *N* (%)	Certain diagnosis* *N* (%)	Probable diagnosis* *N* (%)	TAB performed *N* (%)	TAB positive *N* (%)	Diagnosis based on imaging N (%)	Clinical diagnosis** *N* (%)
1.	Pomeranian (Gdańsk)	67	43 (64)	70 ± 9	24 (36)	24 (36)	35 (52)	32 (48)	6 (9)	3 (50)	34 (51)	27 (45)
2.	West Pomeranian (Szczecin)	48	31 (65)	74 ± 9	22 (46)	23 (48)	39 (81)	9 (19)	21 (46)	15 (71)	43 (90)	0
3.	Masovian (Warszawa) capital city region	43	35 (81)	70 ± 13	25 (58)	21 (49)	30 (70)	13 (30)	2 (5)	1 (50)	24 (56)	17 (42)
	Masovian (Warszawa) site 1	16	14 (88)	71 ± 16	10 (63)	8 (50)	16 (100)	0	0	0	2 (13)	14 (88)
	Masovian (Warszawa) site 2	17	12 (71)	70 ± 13	9 (53)	6 (35)	14 (82)	3 (18)	1 (6)	1 (100)	16 (94)	0
	Masovian (Warszawa) site 3	10	9 (90)	67 ± 10	6 (60)	7 (70)	0	10 (100)	1 (10)	0	7 (70)	2 (30)
4.	Lublin (Lublin)	35	23 (66)	65 ± 14	14 (40)	18 (51)	32 (91)	3 (9)	7 (24)	3 (43)	25 (71)	3 (20)
5.	Łódź (Łódź)	14	12 (86)	73 ± 6	7 (50)	2 (14)	14 (100)	0	2 (17)	2 (100)	1 (7)	11 (79)
6.	Lesser Poland (Kraków)	6	2 (33)	58 ± 9	2 (33)	3 (50)	4 (67)	2 (33)	4 (67)	2 (50)	6 (100)	0
7.	Kuyavian-Pomeranian (Bydgoszcz)	4	3 (75)	71 ± 11	4 (100)	3 (75)	3 (75)	1 (25)	2 (50)	2 (100)	3 (75)	0
8.	Lower Silesian (Wrocław)	1	1 (100)	57	0	1 (100)	1 (100)	0	1 (100)	1 (100)	0	0
9.	Silesian (Katowice)	1	1 (100)	74	0	1 (100)	1 (100)	0	0	0	1 (100)	0
	All	219	151 (69)	70 ± 11	98 (45)	117 (53)	146 (67)	73 (33)	47 (23)	30 (64)	162 (74)	10 (12)

PMR, polymyalgia rheumatica and TAB, temporal artery biopsy. *As per handling clinician’s judgment, **without TAB or imaging.

### High reported prevalence in the regions engaged in the POLVAS registry

Reference hospital centers from all six Voivodeships with the highest reported prevalence according to national insurer data (West Pomeranian, Pomeranian, Masovian, Lower Silesian, Kuyavian-Pomeranian, and Lesser Poland) were participating in the POLVAS registry. Centers from three Voivodeships with the highest reported prevalence (West Pomeranian, Pomeranian, and Masovian) supplied POLVAS with the majority of patients (Table 1). Most of the top recruiting POLVAS centers showed an active interest in GCA and other vasculitides, as 5 of them belonged to founding parties of the POLVAS registry (6). All three actively running fast-track clinics for GCA were localized in centers within the six Voivodeships with the highest reported prevalence. There was a significant correlation between the funding of the fast-track clinics and the number of GCA cases reported per region’s population from 2015—the date when the second center implemented the fast-track approach (Spearman’s rank correlation coefficient, *R* = 0.72, *p* = 0.001). PCA was performed to analyze and detect the overall pattern in the incidence rate throughout the years 2008–2019. After the reduction of multidimensionality, two clusters differentiated institutions where the fast-track approach was implemented separately from the departments without it. Two principal components contribute to 84.9% of variability, implying a strong effect. There was also a high attendance of physicians at GCA ultrasound courses from these sites (defined as at least two attendees per 10^6^ of the population) that were organized in West Pomerania from 2013 to 2017. The site in the region with the highest reported prevalence (West Pomerania) was the first to start fast-track GCA clinic in 2008 and organized annual ultrasound courses on vascular ultrasound in GCA from 2013 to 2017 educating personnel from other centers. It was also one of the founding sites of the POLVAS registry. No fast-track GCA centers were running in 10 Voivodeships with the lowest reported prevalence.

### Differences within the sites engaged in the POLVAS initiative

Diagnostic procedures were analyzed based on the data collected by the centers from nine regions involved in the POLVAS registry, with six regions with the highest reported prevalence among them. There were local differences in the diagnostic procedures applied in the different sites. Imaging was generally used for the diagnosis in 74% of all cases, in contrast with temporal artery biopsy (TAB) performed in 23%. Once performed, TAB yielded 64% of positive results. The site from the region having the highest reported prevalence (West Pomeranian) used arterial visualization for the diagnoses of 90% of patients; however, the percentage of TAB in that site was also relatively high (46% - the third result among all of the centers). The three centers from the capital city region showed different diagnostic approaches: from based on clinical manifestations (88% of patients) and mixed strategy to imaging-based diagnosis (94%), with a low percentage of TAB performed (0–11%). The five POLVAS centers that reported the smallest number of patients (<15 patients per site) in the registry (Łódź, Lesser Poland, Kuyavian-Pomeranian, Lower Silesian, Silesian; all but one coming from the regions with the lowest reported prevalence) included mainly patients with confirmed diagnosis (67–100%) with high number of TAB performed in three of them (50–100%) and high TAB positivity (50–100%). In this group, after excluding sites reporting only one patient, there were two sites (Lublin, Łódź) reporting a high number of patients with certain diagnoses and a low number of patients with a probable diagnosis; however, one of them reported that most of the diagnosis was based on imaging, while the other—on clinical diagnosis (in 79% - the second highest result between all centers).

Polymyalgia rheumatica (PMR) was present in 53% of all patients, and ophthalmological manifestations were present in 45%, which was comparable between the POLVAS sites reporting a high number of cases.

### Treatment

All patients received CSs. They were used in monotherapy in 76% of patients as initial treatment and in 66% during maintenance therapy. The most commonly used disease-modifying antirheumatic drug (DMARD) was methotrexate (MTX)—applied in 18% of patients as initial treatment and in 28% during follow-up. Other DMARDs applied were: cyclophosphamide (CTX), azathioprine (AZA), and mycophenolate mofetil (MMF), but their use was limited to isolated cases. Leflunomide and biologic DMARDs (bDMARDs) were not used. MTX and other DMARDs were mainly used in centers reporting the highest number of patients and in some centers from the capital city region (Table 3).

**Table 3:** Treatment of GCA patients in the POLVAS registry by different centers.

	Voivodship name (location of its reference center)	MTX as initial treatment *N* (%)	DMARD other than MTX as initial treatment *N* (%)	MTX during follow-up treatment* *N* (%)	DMARD other than MTX during follow-up treatment** *N* (%)	CTX any time *N* (%)	AZA any time *N* (%)	MMF any time *N* (%)	LEF and bDMARDs any time
1.	Pomeranian (Gdańsk)	12 (18)	0	23 (34)	0	0	0	1 (1)	0
2.	West Pomeranian (Szczecin)	1 (2)	4 (8)	3 (6)	4 (8)	4 (8)	3 (6)	0	0
3.	Masovian (Warszawa) capital city region	13 (30)	5 (12)	15 (35)	4 (9)	3 (7)	3 (7)	2 (5)	0
	Masovian (Warszawa) site 1	0	0	1 (6)	0	0	0	0	0
	Masovian (Warszawa) site 2	12 (71)	5 (29)	8 (47)	4 (24)	3 (18)	3 (18)	2 (12)	0
	Masovian (Warszawa) site 3	1 (10)	0	6 (60)	0	0	0	0	0
4.	Lublin (Lublin)	15 (43)	0	16 (46)	3 (9)	0	2 (6)	0	0
5.	Łódź (Łódź)	0	0	2 (14)	0	0	0	0	0
6.	Lesser Poland (Kraków)	1 (17)	1 (17)	2 (33)	2 (33)	2 (33)	1 (17)	0	0
7.	Kuyavian-Pomeranian (Bydgoszcz)	0	0	0	0	0	1 (25)	0	0
8.	Lower Silesian (Wrocław)	0	0	0	0	0	0	0	0
9.	Silesian (Katowice)	0	0	0	0	0	0	0	0
	All	42 (19)	10 (5)	61 (28)	13 (6)	9 (4)	10 (5)	3 (1)	0

AZA, azathioprine; bDMARDs, biological disease-modifying antirheumatic drugs; CTX, cyclophosphamide; DMARDs, disease-modifying antirheumatic drugs; LEF, leflunomide; MMF, mycophenolate mofetil; and MTX, methotrexate. *After achieving remission or longer than 6 months.

## Discussion

POLVAS registry is the first large multicenter database of patients with vasculitis in Poland. For this study, especially data with a potential impact on the diagnosis were selected: the presence of ophthalmological manifestations falling within a spectrum of classical temporal arteritis, the presence of PMR, the probability of the diagnosis, and the diagnostic approach—based on biopsy, imaging, or exclusively on clinical manifestations.

In the last few years, an increase in interest in GCA in Poland has become apparent, corresponding with the implementation of fast-track diagnostic pathways and higher rates of reported prevalence, especially in regions containing POLVAS participating centers. Although 219 GCA patients that were included in the POLVAS registry are the largest group thus far described in Poland, it is meaningful that the participants of this registry enrolled as many as 625 adult AAV patients diagnosed up to December 2016 (9). It is reasonable to conclude that reference centers in Poland are still mostly engaged in AAV management and research rather than GCA.

### Demographic data analysis of GCA patients

There was a 10-fold, dynamic, and steady increase in the number of GCA cases reported in Poland between 2008 and 2019. This is a surprisingly high increase, and to our knowledge, a phenomenon not reported previously in vasculitis (3). However, high underdiagnosis of GCA before 2008, demonstrated by very low reported prevalence, may play a role, as there was a start from a very low level. The underdiagnosis was possibly attributable to low awareness of the disease, which was considered an ultra-rare entity in Poland, a lack of local epidemiological analysis on GCA, insufficient education, and a lack of interdisciplinary cooperation, resulting in a lack of referrals to rheumatologists from other specialists. Such a high recent increase in GCA reports is hard to explain exclusively with improvements in the standard of care and healthcare access or changes in population demographics. There were no central campaigns or programs organized in Poland to increase awareness of GCA. Instead, there were regional initiatives of local rheumatology centers with cooperation, bringing improvements in the recognizability that we try to describe in this article. GCA became a frequently discussed topic within rheumatic society, e.g., at local meetings and national conferences (an increase in the number of abstracts on GCA – result not presented) with some spirit of national competition. However, current differences in the reported prevalence of GCA between various regions of Poland are still 10-fold. These differences are unexplained with potential ethnic differences (that are negligible in the uniformly Caucasian Slavic population of Poland) or with local differences in patients’ ancestry that were reported in previous studies (10). These differences are only partially explained by the existence of vasculitis reference centers, which are not formally organized in Poland. Instead, some informal rheumatology reference centers that are localized in the regions’ capitals are well known for their expertise and may recruit more GCA patients.

### High reported prevalence in the regions engaged in the POLVAS registry

Although we analyzed 219 patients included in the POLVAS registry as only a sample of Polish GCA patients, they were mostly reported in the regions with the highest reported prevalence: centers from three Voivodeships with the highest reported prevalence supplied to POLVAS the most of all recruited patients (Table 1). Regions with high rates of reported prevalence had centers that were founding parties of the POLVAS registry or actively recruiting patients in the POLVAS registry and had a running fast-track diagnostic clinic for GCA that significantly corresponded to GCA diagnostic rates. There was also high attendance at GCA ultrasound courses from these sites organized in West Pomerania from 2013 to 2017. Although no formal reference centers devoted to vasculitis exist in Poland, some sites seem to have more interest in GCA, which corresponds to an increase in reported prevalence. The site in the region with the highest reported prevalence (West Pomerania) was the first to start a fast-track clinic in 2008 and organized ultrasound courses on vascular ultrasound in GCA from 2013 to 2017, educating personnel from other centers. It was also among the founding sites of the POLVAS registry and is publishing in the field of GCA (10–12). Establishing and running a fast-track clinic—being a part of the active strategy for GCA diagnosis—corresponds to an increase in diagnostic rates. This process in Poland illustrates the mutual benefit of cooperation by different vasculitis centers in the formal national registry initiatives such as POLVAS in increasing awareness of rare diseases such as GCA and its modern diagnosis and management.

### Differences in sites engaged in the POLVAS initiative

Diagnostic procedures utilized for the diagnosis of GCA were analyzed based on the data collected by the nine centers involved in the POLVAS registry, and there were six regions with the highest reported prevalence among them. From this observation, no causality can be referred to, as both the high reported prevalence and scientific activity can suggest increased interest in GCA. This analysis is limited by sparse data from the regions reporting no patients to the POLVAS registry which corresponded with the lowest reported prevalence. Still, there were local differences in the diagnostic approach between the different sites reflecting different diagnostic strategies. Traditional TAB was performed in only 23% of all the cases while imaging was used for the GCA diagnosis in 74%, implying a modern, imaging-based approach to GCA diagnosis (13) or relatively low popularity or availability of the biopsy. The utilization of imaging for the diagnosis of GCA according to other recent registries’ analysis was even higher, reaching 96% (14). Once performed the general TAB yielded 64% positive results suggesting that the remaining 36% of cases were diagnosed despite negative TAB results. Although 64% positivity seems quite high while there is a general trend to limit TAB for ambiguous cases only, it is still lower than noted in other similar studies (14). The site from the region with the highest reported prevalence (West Pomeranian) utilized artery visualization for 90% of the diagnoses of GCA; however, the percentage of TAB was also high (46% - the third result among all of the centers) implying complementary use of TAB in biopsy positive GCA in this center.

Overall, the diagnostic approaches were quite variable: the differences were obvious even in the three centers from the capital city region: from strongly clinically based diagnosis (88%) and mixed strategy to imaging-based diagnosis (94%), but with a low percent of TAB performed in all of them (0–11%). The low recruiting POLVAS centers from the regions with low reported prevalence included mainly patients with certain but not probable diagnoses (67–100%) with a high number of TAB performed and high TAB positivity, which may imply some potential for future increases in diagnostic rates. Furthermore, in this group, there were large differences according to imaging or clinically based diagnostic strategy.

A comparable number of all patients presented musculoskeletal and ophthalmological manifestations (similarly in the POLVAS sites reporting a high number of cases). PMR was present in 53% of all patients, which is comparable to or slightly higher than reported in previous studies, implying an important role of rheumatologists in diagnosing GCA in patients referred with PMR manifestations (15). It is important in regard to the progressive increase in the annual incidence of PMR associated with GCA that was observed in some studies, e.g., from the northwest of Spain (16). Ophthalmological manifestations were present in 45% of all patients, which is comparable to the previous studies (17) that may suggest well-established cooperation with ophthalmologists.

### Treatment

CSs were widely used in monotherapy both as initial treatment (76% of patients) and during follow-up (66%), which remains a major concern to be improved in the future. Leflunomide and biologic bDMARDs were not reported to be used in GCA in patients from the POLVAS registry. Reported treatment modalities illustrate current limitations in refunding GCA treatment in Poland. However, the limited availability of some drugs seems to be compensated by the use of MTX, which is currently refunded in Poland. MTX was also applied in the induction of remission, although in only 19% of patients. Early MTX induction is a modern approach in accordance with 2021 ACR guidelines (18), although our study was performed between 2008 and 2019—that is before these guidelines were formulated. Early use of MTX might also be also attributed to devotion to MTX being the only refunded DMARD in Poland for this indication and, therefore, the most affordable treatment option for the patient. MTX and other DMARDs were mainly used in the leading centers reporting the highest number of patients and in some centers in the capital city. The use of CTX, AZA, and MMF was reported in only a single case, which is in line with the lack of guidelines or data on their use that may raise concerns about their benefit-to-risk ratio in GCA.

### Limitations

Analyzing diagnostic and therapeutic trends based on registry data is limited by inclusion bias. Our registry data have not been collected in a controlled, subsequent way. This analysis of the data reported to national insurance may not be considered a study to describe actual GCA epidemiology due to the clear underdiagnosis that was illustrated by large regional inequities. GCA is still a rare disease in Poland and is primarily diagnosed in large centers considered reference centers that are responsible for most of the new diagnoses of GCA. This would be a strength of our observation as experienced centers provide trustworthy diagnoses. On the other side, increased interest in GCA might also result in overdiagnosis in the most active sites, although we do not think it would significantly influence the data being the subject of this analysis. We cannot rule out the role of some changes in population demographics and healthcare access on GCA prevalence; however, no such major processes were present in Poland during the study observation period to explain such a high increase in GCA prevalence. GCA cases have been primarily reported to insurance institutions based on the place of diagnosis or performance of further medical procedures and not by the place where patients live; therefore, some large sites considered reference centers, such as the capital city region, may have increased ratios of diagnosis. Conversely, some sparsely populated areas without well-organized reference centers might have decreased ratios of diagnosis. However, after the first diagnosis, the patient should normally return to his place of living to be further reported to insurance locally.

In summary, this is the first multicenter retrospective study of Polish GCA patients, describing the current reported prevalence and underlining regional inequities and diagnostic differences. A substantial increase has been observed in recent years in the diagnostic rates of GCA in Poland. It may be attributed to increased interest accompanied by improved diagnostic modalities with the introduction of fast-track diagnostic pathways in some centers that significantly increased GCA diagnostic rates. However, regional inequities are present. Further cooperation and education (such as implementing targeted educational programs and workshops) are needed to minimize them and unify local diagnostic and therapeutic traditions. This observational study suggests some potential for further increases in the recognizability of GCA and wider use of DMARDs and bDMARDs instead of CS monotherapy. We hope that the Polish experience might be interesting and serve as some guidance for the populations with the problem of underdiagnosis of GCA. Future research should consider prospective data collection to provide more accurate and reliable insights into GCA prevalence and diagnostic practices.

## Data availability statement

The raw data supporting the conclusions of this article will be made available by the authors, without undue reservation.

## Ethics statement

The studies involving humans were approved by Bioethical Commission of Jagiellonian University, decision No. 122.6120.25.2016. The studies were conducted in accordance with the local legislation and institutional requirements. The participants provided their written informed consent to participate in this study.

## Author contributions

MMi: Conceptualization, Formal analysis, Investigation, Methodology, Resources, Writing – original draft, Writing – review & editing. KW: Writing – review & editing. JMu: Writing – review & editing. AM: Writing – review & editing. MMa: Writing – review & editing. RJ: Writing – review & editing. WT: Writing – review & editing. JK-Z: Writing – review & editing. MW: Writing – review & editing. AL-P: Writing – review & editing. JMa: Writing – review & editing. MB: Writing – review & editing.
